# Case report: Neuropathic pain versus undesirable behavior in a Dachshund after hemilaminectomy surgery for an intervertebral disc extrusion

**DOI:** 10.3389/fvets.2023.1223800

**Published:** 2023-07-27

**Authors:** Koen M. Santifort, Marta Plonek, Paul J. J. Mandigers

**Affiliations:** ^1^IVC Evidensia Small Animal Referral Hospital Arnhem, Neurology, Arnhem, Netherlands; ^2^IVC Evidensia Small Animal Referral Hospital Hart van Brabant, Neurology, Waalwijk, Netherlands; ^3^Department of Clinical Sciences, Faculty of Veterinary Medicine, Utrecht University, Utrecht, Netherlands

**Keywords:** canine, neuropathic pain, undesirable behavior, behavioral problem, compulsive disorder

## Abstract

A 5.5 years-old male Dachshund was presented for evaluation because of undesirable behavior including barking, biting, sucking and licking the right-side flank, ventrally and slightly caudally to the level of the surgical incision 7 days after hemilaminectomy for a right-sided L1-2 intervertebral disc extrusion. The dog was being treated with oral gabapentin 10 mg/kg q8h. Repeat clinical examination on three occasions after post-operative discharge did not reveal any signs of hyperesthesia or neurological deficits and the behavior was not observed in the clinic during consultations. During a separate day of hospital admittance with the aim of evaluating for the presence or absence of the behavior, the dog also did not exhibit the behavior. Oral paracetamol 12 mg/kg q8h was added to medical treatment. When the dog was discharged and returned home, the behavior was immediately seen again. When the owners implemented verbal punishment, the behavior immediately ceased. The owner verbally corrected the dogs’ behavior for two excitative days. Upon telephone consultation 3 days later, the owner reported that they only had observed three recurrences of the behavior that immediately ceased following verbal correction and did not recur thereafter. Oral analgesic medication was tapered and discontinued. No recurrence of the behavior was noticed during the next 2 months. The authors postulated the dog possibly expressed signs of neuropathic pain in the post-operative period, or that the behavior was of a “compulsive disorder-like” nature as it only occurred when the dog was at home and in the presence of the owner. The eventual outcome and result of verbal corrections implemented by the owner seem to support the latter. In conclusion, compulsive-like undesirable behavior should be considered a differential diagnosis in dogs in the post-operative period of procedures possibly associated with the development or expression of signs of neuropathic pain.

## Introduction

“Pain” is defined by the International Association for the Study of Pain (IASP) an unpleasant sensory and emotional experience associated with, or resembling that associated with, actual or potential tissue damage (1). Therefore, pain is not something veterinary professionals can necessarily diagnose accurately in their patients. Behavioral signs of pain in dogs may be difficult to interpret as they are purely based on varying clinical signs and we often cannot be sure if the behavior is really due to pain. Neuropathic pain can be specifically enigmatic and difficult to treat (2–4). A common cause of neuropathic pain in dogs is intervertebral disc extrusion (IVDE) (5, 6). In this case report, we present a typical case of a dog with a thoracolumbar IVDE managed surgically and medically, that represented for evaluation of undesirable behavior. A behavior the dog only showed when he was at home with the owners, and which was absent when the dog was evaluated repeatedly in the clinic.

## Case description

A 5.5 years-old male Dachshund was presented for evaluation of recurrent and worsening signs of back pain, paraparesis and proprioceptive ataxia. The patient had started exhibiting signs of back pain including kyphosis and intermittent yelping about 6 weeks prior to presentation. A computed tomographic (CT) study had yielded a diagnosis of a L1-2, right sided intervertebral disc extrusion (IVDE) with spinal cord compression. Since then, the patient had been treated medically for over a month with meloxicam 0.1 mg/kg *per os* (discontinued due to gastrointestinal signs including vomiting and diarrhea), gabapentin 10 mg/kg twice daily and, for the last week, tramadol 3 mg/kg thrice to four times daily. Despite initial positive response, signs of spinal pain persisted and worsened again in the last 2 weeks before presentation.

A general clinical examination revealed no abnormalities. Neurological evaluation revealed ambulatory paraparesis, proprioceptive ataxia, kyphosis and paraspinal hyperesthesia. The neuroanatomical localization was a T3-L3 myelopathy. A high-field magnetic resonance imaging (MRI, 1.5 Tesla, Vantage Elan; Canon Medical Systems, The Netherlands) scan of the thoracolumbar spinal cord was performed to confirm the earlier diagnosis of a L1-2 IVDE and evaluate for other possible causes of the neurological signs (such as IVDE at another site since the earlier CT scan had been performed over a month ago). A right-sided L1-2 IVDE with severe spinal cord compression was confirmed. Surgical treatment was advised and performed (hemilaminectomy on the right side at L1-2). Surgery was uneventful and successful. Perioperative analgesia included methadone (0.2–0.5 mg/kg intravenously every 2–6 h, tapered off over the next 12 h), a bolus of ketamine (5 mg/kg intravenously) followed by a continuous rate infusion of ketamine (5 ug/kg/min intravenously tapered off over the next 24 h), paracetamol (12 mg/kg *per os* every 8 h) and gabapentin (10 mg/kg *per os* every 8 h). The patient recovered well, did not express any signs or behavior consistent with pain and showed significant improvement in ambulatory function (i.e., improving paraparesis and ataxia) during the post-operative hospital stay. The dog had urinated and was ambulatory with minimal ataxia, exhibiting no signs of pain or paraspinal hyperesthesia on palpation of the surgical area. The dog was discharged 1 day following the surgery with oral medication including paracetamol 12 mg/kg q12h for the next 5 days, and gabapentin 10 mg/kg q8h until follow-up scheduled for 2 weeks later. The owners were advised to keep the dog indoors and implement “rest,” and only go out for short walks. The owner reported resolution of all signs at telephone follow-up 2 days later. Four days later, the owner called to express concerns about a possible perianal issue. The dog was exhibiting undesirable behavior including chasing, biting, sucking and licking the right-side flank and groin area (not being able to reach the perianal area). The dog vocalized (barked) profusely when left alone inside the house and turned to the right flank, while biting and licking that area. The dog was presented for a regular clinical neurological post-surgical consult at 7 days post-surgery. The neurological evaluation was unremarkable. The dog was ambulatory without paraparesis or ataxia, non-kyphotic, and exhibited no signs of paraspinal hyperesthesia on palpation. The dog seemed to enjoy rubbing the right-side flank. The perianal region was unremarkable. Anal glands were palpated and expressed with mild pressure yielding a moderate amount of granular grey material (mostly sebum). Owners were instructed to monitor and continue gabapentin as prescribed. Immediately upon returning home, the owner called to say the dog exhibited the undesirable behavior again. A video of the behavior was provided (Supplementary video 1). The video showed the dog barking and biting, sucking and licking the right-side flank, ventrally and slightly caudally to the level of the surgical incision. Owners were instructed to increase the dosage gabapentin (20 mg/kg *per os* every 8 h) based on the speculative possibility of neuropathic pain related to surgical manipulation of the nerve roots at the site of the L1-2 IVDE on the right, consistent with the side where the behavior was directed to. The undesirable behavior, including vocalization (barking), continued. The dog stopped the behavior when the owner would rub the right flank and when he was walked outside, when he was offered food or when he was kept busy otherwise (e.g., playing with a toy). The owners were asked to return to the clinic for another neurological evaluation. They had restarted paracetamol (12 mg/kg *per os* every 8 h) by themselves at this point. Again, the neurological examination was unremarkable. The dog was hospitalized for 24 h for observation, continuing the medication as described. The behavior was not observed during this period in the clinic. The following behavior, deemed to be normal, was seen specifically: the dog was responsive, was enthusiastic when taken for walks, engaged in playing with dog toys, and enjoyed pats on the head and stroking the head, neck and back. After yet another clinical neurological examination, the dog was discharged again the next day and continued on oral medication (paracetamol 12 mg/kg q8h and gabapentin 10 mg/kg q8h). Immediately upon returning home, the dog exhibited the behavior again. The owner now employed verbal correction (“punishment,” i.e., the owner “got mad” and told the dog to stop in a loud voice). The dog immediately stopped the behavior.

The owner expressed the desire to continue to verbally correct the dog if necessary, during the weekend. Upon telephone consultation after the weekend, the owner reported three recurrences of the behavior that were also immediately halted by verbal correction of the owner. Thereafter, the behavior did not recur. Paracetamol and gabapentin were discontinued. No recurrence of the behavior was noticed during the next 2 months.

## Discussion

Neuropathic pain in animals can be difficult to recognize, diagnose, manage and eliminate (2–4). In veterinary neurology and neurosurgery, pathology primarily and physically affecting the nervous system is frequently diagnosed. One of the most common diagnoses is intervertebral disc extrusion (IVDE) (5). A primary clinical sign is “pain” or hyperesthesia (5–7). Paresthesia or dysesthesia are less reliably diagnosed but are expected to occur in these patients as well. A recent study that looked at dogs with thoracolumbar IVDE treated surgically found that as much as 15% of those dogs may experience chronic neuropathic pain (8).

The pathophysiology of neuropathic pain is incompletely understood. It is defined as “pain caused by a lesion or disease of the somatosensory nervous system” (2–4, 9). Pressure on or irritation of a nerve, nerve root, or meninges by any cause can be the underlying issue. One such cause is an IVDE, or “disc herniation” (2–5, 8, 10–14). Neuropathic pain related to IVDE or surgical treatment thereof may, for instance, be related to handling of an already injured/compressed nerve, or the presence of residual compressive material (8, 10–14). Ectopic activity in affected nervous tissue and several other key processes are involved, such as peripheral- and central sensitization, impaired inhibitory neuromodulation, and microglial reactivity (2–4, 15–19). Other important processes include temporal summation, chronicity (influencing peripheral- and central sensitization), (neuro)inflammatory responses, and (neuro)endocrine responses. In human medical literature, psychological, emotional, social, and work-related factors are highlighted as well (20–22). The role thereof in veterinary medicine is difficult, or impossible, to assess.

Taking all of this into account, the authors at first deemed it likely that this dog was experiencing post-operative neuropathic pain after excluding other physical causes of discomfort possibly resulting in behavior suggestive of pain. However, when the behavior was promptly halted after verbal punishment employed by the owners, this raised considerable doubts about that conclusion. Taking into account that the behavior did not recur following verbal correction repeated thrice and that there were no other signs suggestive of neuropathic pain, pain seemed an unlikely cause for the behavioral signs.

Differential diagnoses for the described behavior in this case were perianal or anal gland irritation, neuropathic pain related to the surgery or primary pathology and undesirable behavior (compulsive disorder-like behavior). Perianal and anal gland issues were dismissed after the first clinical follow-up and manual expression of the anal glands, followed by recurrence of the behavior and evaluation of a video provided by the owner. Neuropathic pain was considered likely, since its occurrence in dogs with IVDE and in post-surgical periods is reported (8). However, this specific behavior has not been noted in veterinary literature in dogs treated surgically for IVDE and the involved neurosurgeon had not noticed any such behavior in cases of suspected neuropathic pain (or dysesthesia/paresthesia) before. It would also be atypical or even illogical for neuropathic pain to not be evident clinically and be entirely situation- or environment-dependent. Medical treatment with paracetamol and gabapentin had no effect on the behavior. Finally, undesirable behavior (compulsive disorder-like), unintendedly positively reinforced by the owner at home by giving attention to the dog and rewarding it by rubbing the flank, taking it for walks and feeding were considered a likely option. Immediate cessation of the behavior after verbal punishment by the owner was considered consistent with this latter diagnosis.

In this case, the undesirable behavior was not seen during multiple clinical assessments and only occurred at home. The behavior may have been suppressed during visits to the clinic, e.g., due to stress. Behavior has been shown to be different in dogs when visiting veterinary clinics (23, 24). It may well be that neuropathic pain-related behavior is also differently expressed (or suppressed) in dogs when visiting veterinary clinics compared to being at home. This, as of yet, has not been studied in veterinary medicine. However, in the authors’ opinion, it remains unlikely that neuropathic pain-related behavior would stop so abruptly after verbal correction by the owner as was seen in this case.

Compulsive disorders (CD), or stereotypies, in small animals are characterized by a constant and time-consuming repetition of behaviors that appear to serve no obvious purposes (9, 10). Examples of canine CD include barking, tail chasing, flank sucking, fly catching/biting, circling, toy-chewing and self-mutilation behaviors (25–28). The behavior shown in Supplementary video 1 includes barking and looks like what has been described for flank sucking (28). Tail chasing was considered, though the tail does not seem to be the object of chase in this case. Luescher stated that “because a CD derives from conflict behavior, an attempt should be made to identify and remove the cause of conflict, frustration, and stress.” (26). In the case reported here, it may be that the source of stress was the implemented “rest” by the owners. The dog was used to frequent walks and playing. The lack thereof might have been a source of frustration. Indeed, according to Luescher, an important stress-inducing factor is the lack of predictability and control over the environment. “Inconsistent owner-animal interaction” is specifically mentioned as an example.

For canine CD, various treatment modalities including training methods and medications have been reported (25–28). Verbal correction (or punishment, “scolding”) is frequently implemented by owners (26, 29). Generally, this is not recommended by veterinary behavioral specialists for a number of reasons (26, 29), including the inability to apply owner-related punishment correctly and consistently in the majority of cases. In this case, the behavior was specifically related to the owner being present, which facilitated the owners’ ability to apply the verbal correction consistently when the dog exhibited the behavior for the three subsequent times. After that, the behavior was not repeated by the dog.

This swift amelioration of the undesirable behavior in this case after verbal correction by the owners was remarkable. To the authors, this suggests that the term CD may not yet have been applicable to this case, although there is no is no specific set time or number of repetitions for an undesirable behavior to be classified as a CD. The term “compulsive disorder-like” was discussed with the owners. Still, a true CD would be unlikely to resolve after just four bouts (total) of scolding by an owner. For CD, it is deemed of vital importance to start treatment as early on as possible as outcome is negatively affected by problem duration (26).

Limitations to this case report include the lack of a follow-up MRI study to evaluate for the level of decompression and possible post-operative complications and the lack of quantitative sensory assessments (8). The complete lack of signs of hyperesthesia on clinical examinations post-surgery when the dog was presented for evaluation for the undesirable behavior did not merit such procedures at that time. Consultation with a veterinary behavioral specialist for interdisciplinary management strategy evaluations was considered, but the problem was resolved before the authors did so. Such interdisciplinary approaches should be kept in mind for future cases (25).

In conclusion, not every dog that undergoes surgery for the treatment of thoracolumbar IVDE expressing signs possibly referable to neuropathic pain in the post-operative period may, indeed, be experiencing neuropathic pain. Undesirable behavior (“compulsive disorder-like”) should be considered a differential diagnosis for dog exhibiting constant and repetitive behavior in the post-operative period.

## Data availability statement

The original contributions presented in the study are included in the article/Supplementary material, further inquiries can be directed to the corresponding author.

## Ethics statement

Ethical review and approval was not required for the animal study because the animal was treated in accordance with the local legislation and institutional requirements. Written informed consent was obtained from the owners for the participation of their animals in this study.

## Author contributions

KS contributed to management of the case and performed the surgery and wrote the first draft of the manuscript. KS, MP, and PM evaluated and discussed the video provided by the owner and participated in the revision of the manuscript. All authors contributed to the article and approved the submitted version.

## Funding

The publication fee was covered by IVC Evidensia’s fund for publication of peer-reviewed scientific articles.

## Conflict of interest

The authors declare that the research was conducted in the absence of any commercial or financial relationships that could be construed as a potential conflict of interest.

## Publisher’s note

All claims expressed in this article are solely those of the authors and do not necessarily represent those of their affiliated organizations, or those of the publisher, the editors and the reviewers. Any product that may be evaluated in this article, or claim that may be made by its manufacturer, is not guaranteed or endorsed by the publisher.

## References

[1] International Association for the Study of Pain (ISAP). Classification of chronic pain. 2nd ed. Washington, DC: International Association for the Study of Pain (2023). Available at: https://www.iasp-pain.org/resources/terminology/#pain.

[2] Grubb T. Chronic neuropathic pain in veterinary patients. Top Companion Anim Med. (2010) 25:45–52. doi: 10.1053/j.tcam.2009.10.007, PMID: 20188338

[3] Mathews KA. Neuropathic pain in dogs and cats: if only they could tell us if they hurt. Vet Clin North Am Small Anim Pract. (2008) 38:1365–414. doi: 10.1016/j.cvsm.2008.09.001, PMID: 18954689

[4] Moore SA. Managing neuropathic pain in dogs. Front Vet Sci. (2016) 3:12. doi: 10.3389/fvets.2016.00012, PMID: 26942185 PMC4762016

[5] Fenn J Olby NJ Canine Spinal Cord Injury Consortium (CANSORT-SCI). Classification of intervertebral disc disease. Front Vet Sci. (2020) 7:579025. doi: 10.3389/fvets.2020.579025, PMID: 33134360 PMC7572860

[6] Thompson K Moore S Tang S Wiet M Purmessur D. The chondrodystrophic dog: a clinically relevant intermediate-sized animal model for the study of intervertebral disc-associated spinal pain. JOR Spine. (2018) 1:e1011. doi: 10.1002/jsp2.1011, PMID: 29984354 PMC6018624

[7] Moore SA Tipold A Olby NJ Stein V Granger N Canine Spinal Cord Injury Consortium (CANSORT-SCI). Current approaches to the management of acute thoracolumbar disc extrusion in dogs. Front Vet Sci. (2020) 7:610. doi: 10.3389/fvets.2020.00610, PMID: 33117847 PMC7521156

[8] Zidan N Medland J Olby N. Long-term postoperative pain evaluation in dogs with thoracolumbar intervertebral disk herniation after hemilaminectomy. J Vet Intern Med. (2020) 34:1547–55. doi: 10.1111/jvim.15800, PMID: 32462728 PMC7379041

[9] International association for the study of pain (ISAP). Neuropathic pain special interest group (NeuPSIG). Washington, DC: International Association for the Study of Pain (2023). Available at: https://www.iasp-pain.org/group/neuropathic-pain-neupsig/.

[10] Baron R Binder A Attal N Casale R Dickenson AH Treede RD. Neuropathic low back pain in clinical practice. Eur J Pain. (2016) 20:861–73. doi: 10.1002/ejp.838, PMID: 26935254 PMC5069616

[11] Hasvik E Haugen AJ Gjerstad J Grøvle L. Assessing neuropathic pain in patients with low back-related leg pain: comparing the painDETECT questionnaire with the 2016 NeuPSIG grading system. Eur J Pain. (2018) 22:1160–9. doi: 10.1002/ejp.1204, PMID: 29436056

[12] Jensen RK Kongsted A Kjaer P Koes B. Diagnosis and treatment of sciatica. BMJ. (2019) 367:l6273. doi: 10.1136/bmj.l6273 31744805

[13] Patel EA Perloff MD. Radicular pain syndromes: cervical, lumbar, and spinal stenosis. Semin Neurol. (2018) 38:634–9. doi: 10.1055/s-0038-1673680, PMID: 30522138

[14] Samuelly-Leichtag G Eisenberg E Zohar Y Andraous M Eran A Sviri GE. Mechanism underlying painful radiculopathy in patients with lumbar disc herniation. Eur J Pain. (2022) 26:1269–81. doi: 10.1002/ejp.1947, PMID: 35357731 PMC10083974

[15] Baron R Binder A Wasner G. Neuropathic pain: diagnosis, pathophysiological mechanisms, and treatment. Lancet Neurol. (2010) 9:807–19. doi: 10.1016/S1474-4422(10)70143-5 20650402

[16] Eide PK. Wind-up and the NMDA receptor complex from a clinical perspective. Eur J Pain. (2000) 4:5–15. doi: 10.1053/eujp.1999.0154, PMID: 10833550

[17] Eisenberg E Burstein Y Suzan E Treister R Aviram J. Spinal cord stimulation attenuates temporal summation in patients with neuropathic pain. Pain. (2015) 156:381–5. doi: 10.1097/01.j.pain.0000460342.69718.a2, PMID: 25599230

[18] Imamura Y Shinozaki T Okada-Ogawa A Noma N Shinoda M Iwata K. An updated review on pathophysiology and management of burning mouth syndrome with endocrinological, psychological and neuropathic perspectives. J Oral Rehabil. (2019) 46:574–87. doi: 10.1111/joor.12795, PMID: 30892737

[19] Sommer C Leinders M Üçeyler N. Inflammation in the pathophysiology of neuropathic pain. Pain. (2018) 159:595–602. doi: 10.1097/j.pain.0000000000001122 29447138

[20] Haythornthwaite JA Benrud-Larson LM. Psychological aspects of neuropathic pain. Clin J Pain. (2000) 16:S101–5. doi: 10.1097/00002508-200006001-00017 10870748

[21] Malik T Malik A Abd-Elsayed A. Pathophysiology of work-related neuropathies. Biomedicine. (2023) 11:1745. doi: 10.3390/biomedicines11061745, PMID: 37371842 PMC10296724

[22] Torta R Ieraci V Zizzi F. A review of the emotional aspects of neuropathic pain: from comorbidity to co-pathogenesis. Pain Ther. (2017) 6:11–7. doi: 10.1007/s40122-017-0088-z, PMID: 29178035 PMC5701895

[23] Lind AK Hydbring-Sandberg E Forkman B Keeling LJ. Assessing stress in dogs during a visit to the veterinary clinic: correlations between dog behavior in standardized tests and assessments by veterinary staff and owners. J Vet Behav. (2017) 17:24–31. doi: 10.1016/j.jveb.2016.10.003

[24] Mariti C Raspanti E Zilocchi M Carlone B Gazzano A. The assessment of dog welfare in the waiting room of a veterinary clinic. Anim Welf. (2015) 24:299–305. doi: 10.7120/09627286.24.3.299

[25] d'Angelo D Sacchettino L Carpentieri R Avallone L Gatta C Napolitano F. An interdisciplinary approach for compulsive behavior in dogs: a case report. Front Vet Sci. (2022) 9:801636. doi: 10.3389/fvets.2022.801636, PMID: 35400099 PMC8988433

[26] Luescher AU. Diagnosis and management of compulsive disorders in dogs and cats. Vet Clin North Am Small Anim Pract. (2003) 33:253–67. doi: 10.1016/S0195-5616(02)00100-6, PMID: 12701511

[27] Tiira K Hakosalo O Kareinen L Thomas A Hielm-Björkman A Escriou C. Environmental effects on compulsive tail chasing in dogs. PLoS One. (2012) 7:e41684. doi: 10.1371/journal.pone.0041684, PMID: 22844513 PMC3406045

[28] Moon-Fanelli AA Dodman NH Cottam N. Blanket and flank sucking in Doberman pinschers. J Am Vet Med Assoc. (2007) 231:907–12. doi: 10.2460/javma.231.6.907, PMID: 17867975

[29] Blackwell EJ Twells C Seawright A Casey RA. The relationship between training methods and the occurrence of behavior problems, as reported by owners, in a population of domestic dogs. J Vet Behav. (2008) 3:207–17. doi: 10.1016/j.jveb.2007.10.008

